# Distinct Carbon and Nitrogen Metabolism of Two Contrasting Poplar Species in Response to Different N Supply Levels

**DOI:** 10.3390/ijms19082302

**Published:** 2018-08-06

**Authors:** Sen Meng, Shu Wang, Jine Quan, Wanlong Su, Conglong Lian, Dongli Wang, Xinli Xia, Weilun Yin

**Affiliations:** 1 Beijing Advanced Innovation Center for Tree Breeding by Molecular Design, National Engineering Laboratory for Tree Breeding, College of Biological Sciences and Technology, Beijing Forestry University, Beijing 100083, China; mengsen021124@126.com (S.M.); saphena.wang@163.com (S.W.); bjfususan@163.com (W.S.); liancl00@163.com (C.L.); wangdongli1997@163.com (D.W.); xiaxl@bjfu.edu.cn (X.X.); 2 College of Forestry, Henan Agricultural University, Zhengzhou 450002, China; quanjine@gmail.com

**Keywords:** nitrogen uptake, photosynthate allocation, transcriptional regulation, stable nitrogen isotope, net inorganic nitrogen flux

## Abstract

Poplars have evolved various strategies to optimize acclimation responses to environmental conditions. However, how poplars balance growth and nitrogen deficiency remains to be elucidated. In the present study, changes in root development, carbon and nitrogen physiology, and the transcript abundance of associated genes were investigated in slow-growing *Populus simonii* (Ps) and fast-growing *Populus euramericana* (Pe) saplings treated with low, medium, and high nitrogen supply. The slow-growing Ps showed a flourishing system, higher δ^15^N, accelerated C export, lower N uptake and assimilation, and less sensitive transcriptional regulation in response to low N supply. The slow-growing Ps also had greater resistance to N deficiency due to the transport of photosynthate to the roots and the stimulation of root development, which allows survival. To support its rapid metabolism and growth, compared with the slow-growing Ps, the fast-growing Pe showed greater root development, C/N uptake and assimilation capacity, and more responsive transcriptional regulation with greater N supply. These data suggest that poplars can differentially manage C/N metabolism and photosynthate allocation under different N supply conditions.

## 1. Introduction

Due to the dual pressure of environmental problems and energy issues, forestation has become critical to prevent soil erosion and desertification, regulate climate, mitigate CO_2_ emissions, and generate bioenergy resources. China is the country possessing the largest artificial forest in the world [1]. Considering the timber production and environmental adaptability, plantations in North China mainly rely on poplars. The genus *Populus* consists of 30–40 species that have been widely used for afforestation in temperate and boreal regions [2]. However, fertile soils in plains areas are often intensively used for agriculture to meet food demands. Poplar plantations have often been established on marginal lands and, therefore, suffer from N deficiency [3]. In this context, it is particularly important to better understand the responses of *Populus* to different N supply levels and to select poplar species that tolerate N deficiency and/or N fertilizer. High biomass and a short coppicing rotation make poplar an important tree species for the paper industry, biofuels, and ecological environmental protection [4]. Many studies have indicated that a sufficient N supply induces greater photosynthesis, greater biomass production, and accelerated C/N metabolism in plants [5,6,7]. For instance, the responses of the belowground biomass and lignin to high N supply are more pronounced in *Populus tremula* × *Populus tremuloides* than in *Populus trichocarpa* [5]. N-fertilization increased the photosynthetic rate and nitrogen use efficiency of *Populus alba × Populus glandulosa* more than *Populus popularis* [8]. These results indicate that the genus *Populus* contains dozens of species that show varied C/N metabolism and molecular regulation in response to varying N availability. However, it remains to be elucidated how poplars that differ in their tolerance to low N supply balance growth and C/N physiology, and the selection of poplar species adapted to particular afforestation site conditions is of particular interest. 

**Figure 1:**
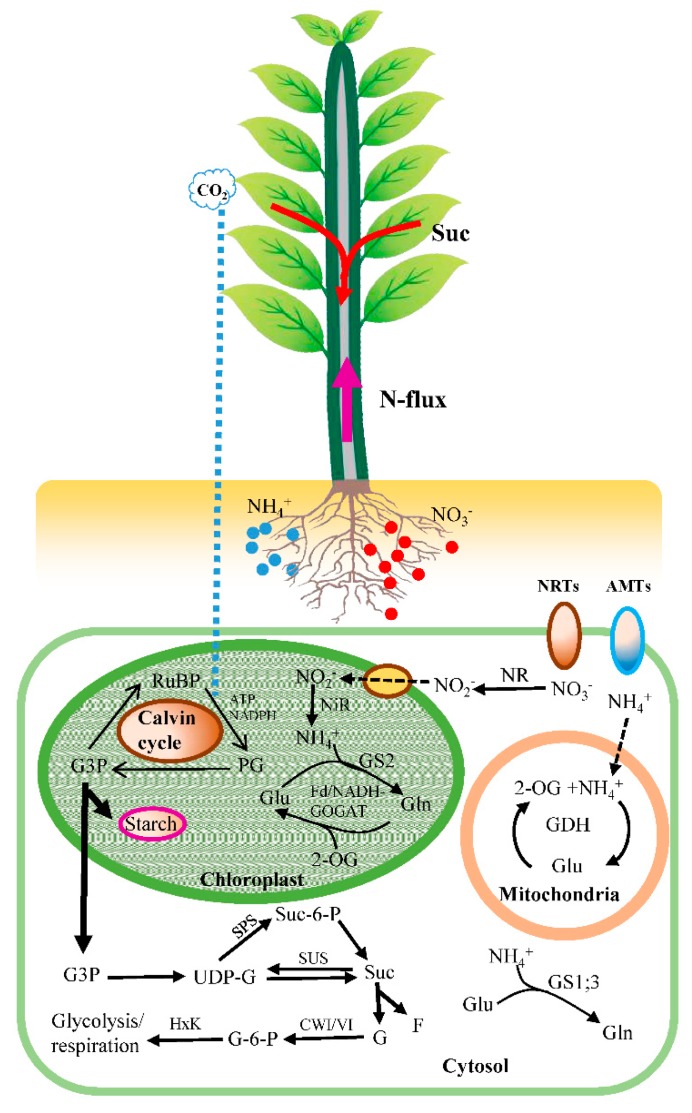
A schematic model of C/N metabolism in plants. Glyceraldehyde 3-phosphate (G3P) produced by CO_2_ fixation in Calvin cycle can be exported to the cytosol or used to synthesize starch in chloroplast. Sucrose is synthesized by the enzyme sucrose phosphate synthase (SPS) via the consumption of uridine diphosphate glucose (UDP-G) from the Calvin cycle and fructose-6-phosphate. Sucrose can be transported to the sink tissues and hydrolyzed to glucose and fructose by cell wall invertase (CWI) and vacuolar invertase (VI). The glucose is phosphorylated by hexokinases (HxKs) and further utilized for glycolysis and respiration. NH_4_^+^ and NO_3_^−^ are absorbed in the roots by various transporters for ammonium (*AMTs*) and nitrate (*NRTs*). After uptake, NO_3_^−^ is reduced to NH_4_^+^ via nitrate reductase (NR) and nitrite reductase (NiR). Then, NH_4_^+^ is assimilated to glutamine (Gln) and glutamate (Glu) by glutamine synthetase and glutamate synthase (GS/GOGAT) or the glutamate dehydrogenase (GDH) pathways.

C metabolism in plants primarily includes carbon fixation, carbohydrate anabolism and carbohydrate utilization. Related to these processes, sucrose plays pivotal roles in plant growth, metabolism regulation, and stress resistance [9]. Sucrose influences and reflects photosynthesis, C export, and nutrient mobilization (Figure 1). Sucrose is the primary product of photosynthesis, the form in which carbohydrates are transported from the photosynthetic tissues (i.e., the source) to non-photosynthetic tissues (i.e., the sink) and the initial building block of most natural organic matter [10]. In the photosynthetic tissues, sucrose is synthesized by the enzyme sucrose phosphate synthase (SPS) via the consumption of uridine diphosphate glucose (UDP-G) from the Calvin cycle and fructose-6-phosphate. Then, sucrose-6-phosphate phosphatase (SPP) hydrolyzes sucrose-6-phosphate into sucrose (Figure 1) [11]. Sucrose can be exported to the apoplast and transported into the sieve element via the plasmodesmata by sucrose transporter (SUT) proteins [12]. Subsequently, turgor pressure provides the driving force for the long-distance transport of sucrose from the source to the sink tissues. Sucrose can be hydrolyzed in the sink tissues to glucose and fructose by cell wall invertase (CWI) and vacuolar invertase (VI). The glucose is phosphorylated by hexokinases (HxKs) and further utilized for glycolysis and respiration. However, comparisons of C metabolism and photosynthate partitioning in poplars that show large differences in growth remain scarce.

Plants can use a wide range of N forms, ranging from simple inorganic N compounds (ammonium and nitrate) to organic N, such as proteins. Many studies have provided evidence for plant uptake of organic N in the field but direct evidence that organic N contributes significantly to plant N nutrition is still lacking [13]. Thus, research on plant N nutrition has had a strong focus on inorganic N forms [13]. Ammonium (NH_4_^+^) and nitrate (NO_3_^−^) are two major forms of inorganic N available for plants in soil [14]. N metabolism in plants primarily includes NH_4_^+^/NO_3_^−^ uptake, transport, and assimilation for amino acid synthesis (Figure 1). NH_4_^+^ and NO_3_^−^ are absorbed in the roots by various transporters for ammonium (*AMTs*) and nitrate (*NRTs*) and form ion fluxes at the root surface. The genome of *Populus trichocarpa* has been documented to contain 14 putative *AMTs* and 79 putative *NRTs* [15]. A few studies have indicated that transcript levels of *AMTs* and *NRTs* (e.g., *AMT1.2*, *AMT1.6*, *NRT1.1*, *NRT2.4B*, *NRT2.4C*, and *NRT3.1C*) were responsive to the environmental N supply. A non-invasive micro-test technique (NMT), a powerful tool in the investigation of in situ ion fluxes, has been widely used to explore the electrophysiological processes of nitrogen acquisition at the root surface [16]. Spatial and temporal dynamics of net ion fluxes and influences of environmental factors such as pH in the media on net ion fluxes have been reported for roots of maize, barley, rice, poplar, and species of eucalyptus and coniferous [3,17,18,19]. Additionally, many studies assume that ^15^N at natural abundance levels acts as a tracer, and significant discrimination has been observed for plants [20]. Discrimination is positive in most biological systems; therefore, the product has a lower δ^15^N value than the substrate [21]. After uptake, NO_3_^−^ is reduced to NH_4_^+^ via nitrate reductase (NR) and nitrite reductase (NiR). Then, NH_4_^+^ is assimilated to glutamine and glutamate by glutamine synthetase and glutamate synthase (GS/GOGAT) or the glutamate dehydrogenase (GDH) pathways (Figure 1). Glutamine and glutamate are utilized as precursors to synthesize various amino acids and proteins. To date, however, few studies have investigated the NH_4_^+^/NO_3_^−^ uptake and assimilation response to the external N supply to compare N metabolism in different poplar species under different environmental conditions.

Little information is available about the C/N metabolism of various poplar species and how they balance growth and nutrient mobilization in the context of a variable N supply. *Populus simonii* (Ps) is widely distributed on the Loess Plateau, whereas *Populus euramericana* (Pe) ordinarily grows on the plains. Soils on the Loess Plateau in Northwest China are alkaline (pH 8–9) and low in available N [22,23,24]. However, Pe grows in the plains soil with sufficient available N. In our previous study, we showed that the slow-growing Ps had greater root growth and NH_4_^+^ assimilation under N-deficient conditions compared to plants with an unlimited N supply, indicating that the plants acclimated to a limiting N supply by allocating more carbon to the root system [23]. However, in another preliminary experiment, the fast-growing Pe was sensitive to low N supply and showed growth inhibition in both the belowground and aerial parts. To identify the differences in C/N physiology in two poplar species with different growth characteristics and elucidate the mechanism behind these phenomena, Ps and Pe saplings were exposed to low and sufficient levels of N. We examined changes in the morphology (i.e., root characteristics), physiology (i.e., photosynthesis, C/N metabolism), and molecular characteristics (i.e., levels of transcripts of representative genes involved in C/N metabolism) in response to different N levels. We hypothesized that (1) the slow-growing Ps can maintain high N uptake and assimilation and prioritizes resource allocation to the roots under low nitrogen supply to adapt to the N deficiency; and (2) an accelerated C/N metabolism in Pe with a sufficient N supply results in fast growth and rapid biomass accumulation.

## 2. Results

### 2.1. Root Growth and Photosynthetic Characteristics

The total root length, root surface and root volume were increased in Ps, but decreased in Pe with low N supply compared to the condition of medium and high N supply (Table 1). Ps also showed higher photosynthesis than Pe under low N supply (Table 1). However, the chlorophyll content and net photosynthetic rate were similar in Ps and Pe with medium and high N supply. Thus, low N supply also led to greater reductions in photosynthesis in Pe than in Ps (Table 1 and Table S3).

### 2.2. Contents of Sucrose, Fructose, Glucose, and Total Carbon

**Figure 2:**
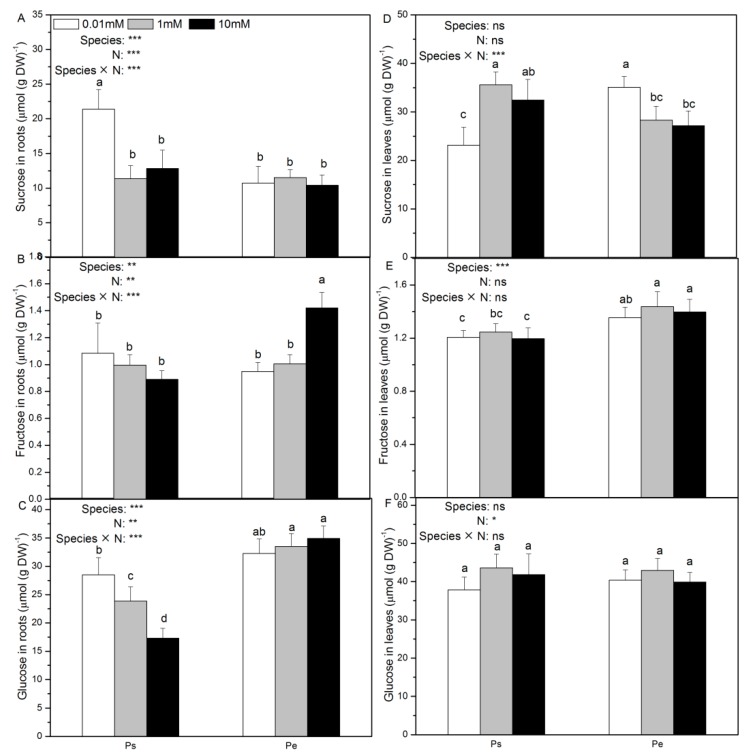
Contents of sucrose (**A**,**D**), fructose (**B**,**E**) and glucose (**C**,**F**) of *P. simonii* (Ps) and *Populus euramericana* (Pe) under 0.01, 1, and 10 mM NH_4_NO_3_. Bars labelled with different letters indicate significant difference between the treatments. *p*-Values of the ANOVAs of species, N treatment, and their interaction are indicated. * *p* < 0.05; ** *p* < 0.01; *** *p* < 0.001; ns, not significant.

Low N supply induced higher root sucrose, glucose and total carbon contents in Ps, but had no impact on these parameters in Pe (Figure 2A,C and Figure S2). In Ps, root fructose content remained unchanged by the tested N levels (Figure 2B). However, higher root fructose content was observed with increasing N supply in Pe (Figure 2B). Foliar sucrose and total carbon content decreased in Ps in response to low N supply (Figure 2D). In both species, the N supply had no effect on foliar fructose or glucose content (Figure 2E,F). 

Generally, sucrose, fructose, and glucose contents in roots and leaves were higher or similar in Pe than in Ps at given three N levels, with the exception that root sucrose content was higher in Ps than in Pe with low N supply (Figure 2).

### 2.3. Root and Leaf Activities of Enzymes Involved in C Assimilation

**Figure 3:**
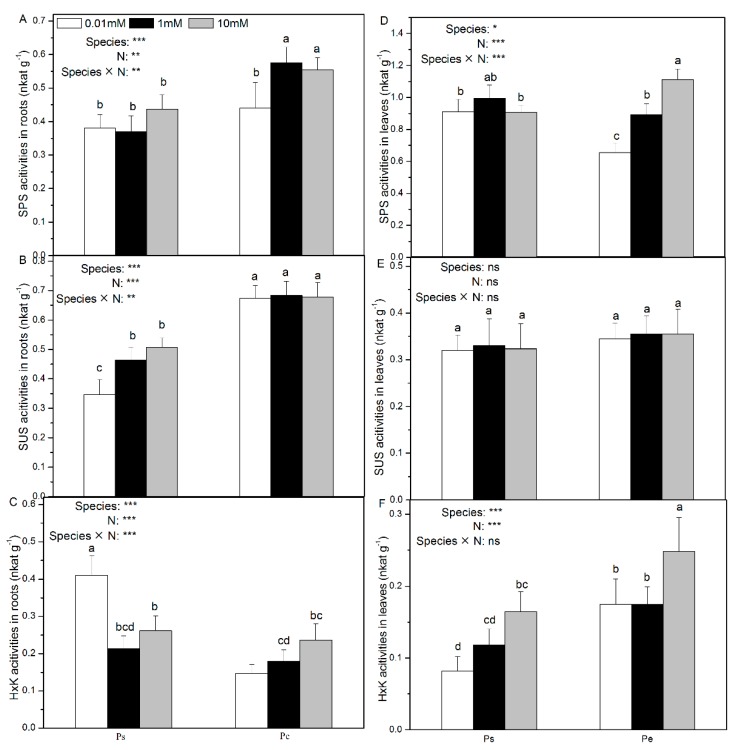
Sucrose phosphate synthase (**A**,**D**), sucrose synthase (**B**,**E**), and hexokinase (**C**,**F**) activities of *P. simonii* (Ps) and *Populus euramericana* (Pe) under 0.01, 1, and 10 mM NH_4_NO_3_. Bars labelled with different letters indicate significant difference between the treatments. *p*-Values of the ANOVAs of species, N treatment, and their interaction are indicated. * *p* < 0.05; ** *p* < 0.01; *** *p* < 0.001; ns, not significant.

In Ps, low N supply had no effect on the root and foliar SPS activities (Figure 3A,C). However, in Pe, the SPS activities in both roots and leaves were stimulated by high N supply (Figure 3A,C). The root SUS activity was increased with increasing N supply in Ps but remained unaltered in Pe (Figure 3B). The foliar sucrose synthase (SUS) activity was also unaffected by N availability in both Ps and Pe (Figure 3D). The HxK activity in the roots was inhibited in Ps by high N supply, but the opposite pattern was observed in Pe (Figure 3E). However, the HxK activity in the leaves was increased by high N supply in both species (Figure 3F). Generally, with medium and high N supply, Ps showed lower SPS activity than Pe (Figure 3A). However, with low N supply, the foliar SPS activity was higher in Ps than in Pe (Figure 3B). The SUS activity in roots was lower in Ps than in Pe at all three N levels, and the SUS activity in leaves was similar in the two poplar species (Figure 3D). At all three N levels, the foliar HxK activity in Pe was higher than in Ps (Figure 3F).

The root CWI activity was not affected by the N supply in Ps, but was increased by high N supply in Pe (Figure S3). In Ps, the root VI activity decreased with high N supply, but in Pe, it was not affected by N supply (Figure S3). Low N supply induced a higher foliar CWI activity in Ps (Figure S3). The foliar VI activity was also unaffected by the N supply (Figure S3). The CWI and VI activities were generally lower in Ps than in Pe, especially with medium and high N supply (Figure S3). However, under low N supply, the foliar CWI activity was higher in Ps than Pe (Figure S3).

### 2.4. Net Fluxes of NH_4_^+^, NO_3_^–^, and H^+^; Accumulation of NH_4_^+^, NO_3_^−^, and NO_2_^−^; and δ^15^N Content

**Figure 4:**
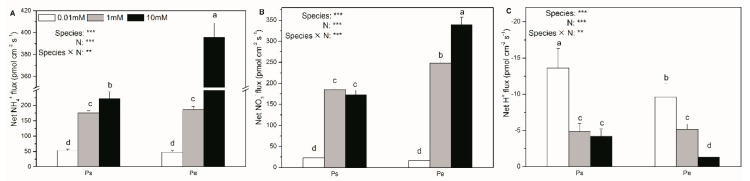
Net NH_4_^+^ (**A**), NO_3_^−^ (**B**) and H^+^ (**C**) fluxes of *P. simonii* (Ps) and *Populus euramericana* (Pe) under 0.01, 1 and 10 mM NH_4_NO_3_. Bars labelled with different letters indicate significant difference between the treatments. *p*-Values of the ANOVAs of species, N treatment, and their interaction are indicated. ** *p* < 0.01; *** *p* < 0.001; ns, not significant.

Generally, in both Ps and Pe, the net NH_4_^+^ and NO_3_^−^ fluxes were significantly decreased by N deficiency (Figure 4A,B and Figure S4). In contrast, both species showed increases in the net H^+^ flux in response to a low N supply (Figure 4C). With low and medium N supply, Ps and Pe exhibited a similar net NH_4_^+^ flux (Figure 4A). However, with high N supply, the net NH_4_^+^ flux was greater in Pe than in Ps (Figure 4A). With low N supply, the net NO_3_^−^ flux was similar between the two species, but with high N supply, it was higher in Pe than in Ps (Figure 4B). With both low and high N supply, net H^+^ flux was higher in Ps than in Pe (Figure 4C). 

**Figure 5:**
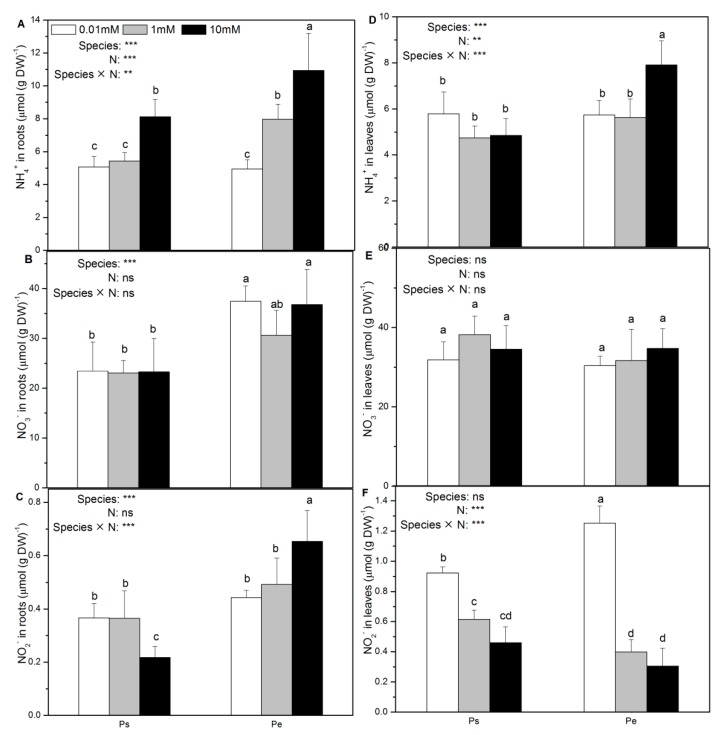
NH_4_^+^ (**A**,**D**), NO_3_^−^ (**B**,**E**), and NO_2_^−^ (**C**,**F**) concentrations of *P. simonii* (Ps) and *Populus euramericana* (Pe) under 0.01, 1, and 10 mM NH_4_NO_3_. Bars labelled with different letters indicate significant difference between the treatments. *p*-Values of the ANOVAs of species, N treatment, and their interaction are indicated. ** *p* < 0.01; *** *p* < 0.001; ns, not significant.

In both species, the root NH_4_^+^ concentration decreased with decreasing N supply levels (Figure 5A). Further, in both poplar species, the N supply had no effect on the NO_3_^−^ concentration in roots and leaves (Figure 5B,E). The NO_2_^−^ concentration in the roots decreased in Ps but increased in Pe in response to high N supply (Figure 5C). However, the foliar NH_4_^+^ concentration increased in Pe in response to high N supply (Figure 5D). Low N supply increased the foliar NO_2_^−^ concentration in both Ps and Pe (Figure 5F). Concentrations of NH_4_^+^, NO_3_^−^ and NO_2_^−^ in roots and leaves were generally similar between the species or lower in Ps than in Pe, especially with high N supply (Figure 5). 

**Figure 6:**
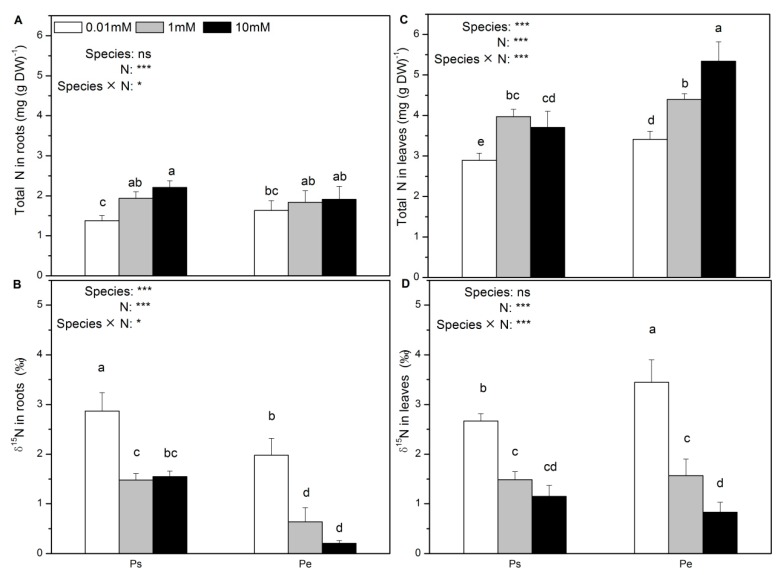
Total N concentrations (**A**,**C**) and δ^15^N (**B**,**D**) of *P. simonii* (Ps) and *Populus euramericana* (Pe) under 0.01, 1, and 10 mM NH_4_NO_3_. Bars labelled with different letters indicate significant difference between the treatments. *p*-Values of the ANOVAs of species, N treatment, and their interaction are indicated. * *p* < 0.05; *** *p* < 0.001; ns, not significant.

Low N supply decreased the total N concentration in the roots and leaves in both species (Figure 6A,C). By contrast, the δ^15^N in roots and leaves was decreased with an increased N supply in both Ps and Pe (Figure 6B,D). The total N concentration in the roots was unaffected by species (Figure 6A). At all three N levels, the level of δ^15^N in the roots was higher in Ps than in Pe (Figure 6B). Generally, compared to Ps, Pe had a higher foliar N concentration but lower foliar δ^15^N (Figure 6C,D). 

### 2.5. Activities of Enzymes in Roots and Leaves Involved in N Assimilation

**Figure 7:**
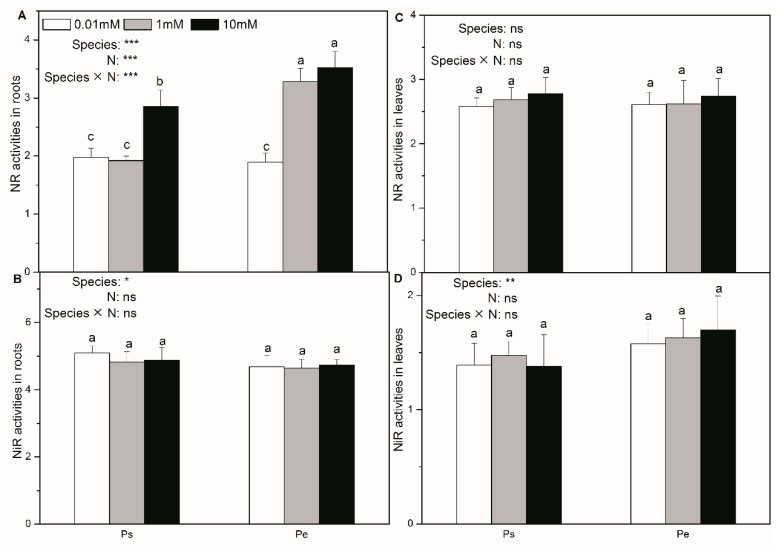
NR (**A**,**C**) and NiR (**B**,**D**) activities of *P. simonii* (Ps) and *Populus euramericana* (Pe) under 0.01, 1, and 10 mM NH_4_NO_3_. Bars labelled with different letters indicate significant difference between the treatments. *p*-Values of the ANOVAs of species, N treatment, and their interaction are indicated. * *p* < 0.05; ** *p* < 0.01; *** *p* < 0.001; ns, not significant.

In both Ps and Pe, NR activity in roots was inhibited by low N supply (Figure 7A). However, N supply did not affect the root NiR activity, the foliar NR activity, or the foliar NiR activity in either species (Figure 7B–D). With a high N supply, Pe had a higher root NR activity than Ps (Figure 7A). The root NiR activity, the foliar NR activity and the foliar NiR activity were similar between the two poplars with the same N supply (Figure 7B–D). 

N supply levels had little effect on GS, GOGAT, and GDH activities (except foliar GS activities) in Pe (Figure S4). However, low N supply generally induced higher foliar GS, GOGAT, and GDH activities in Ps (Figure S4). With medium and high N supply, GS, GOGAT, and GDH activities in roots and leaves were generally lower in Ps than in Pe (Figure S4). By contrast, under low N supply, Ps showed activities of NH_4_^+^ assimilation enzymes that were similar to, or even higher than in Pe (Figure S4).

### 2.6. PCA of Root Characteristics and C/N Physiological Responses

**Figure 8:**
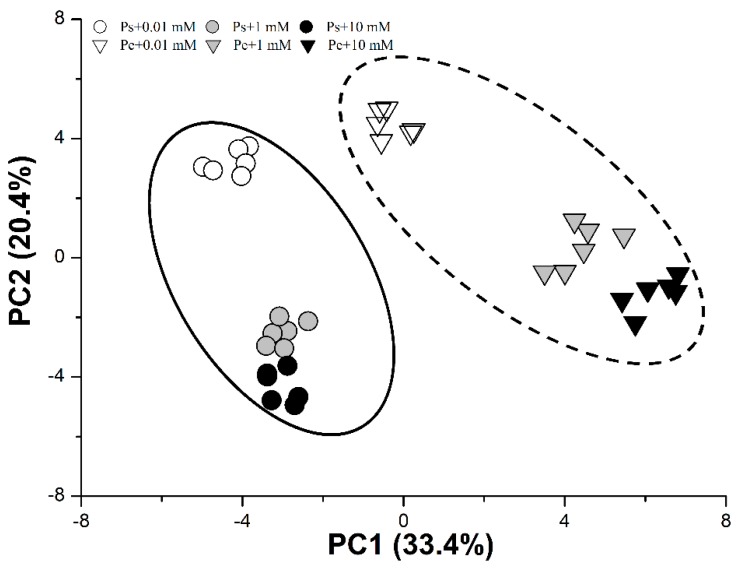
Principal component analysis (PCA) plot of the first two principal components in *P. simonii* (Ps) and *Populus euramericana* (Pe) under 0.01, 1, and 10 mM NH_4_NO_3_. The analysis was conducted using data of physiological parameters of Ps (circle) and Pe (triangle).

PC1 and PC2 accounted for 33.4% and 20.4% of the variation, respectively (Figure 8 and Table S2). PC1 uncoupled the effect of species, and PC2 clearly separated the variation due to N supply. Root CWI activities, root surface area, and δ^15^N in roots were key contributors to PC1, whereas foliar ^15^N and chlorophyll contents were important factors influencing PC2 (Figure 8).

### 2.7. Transcript Levels of Key Genes Involved in C/N Metabolisms

**Figure 9:**
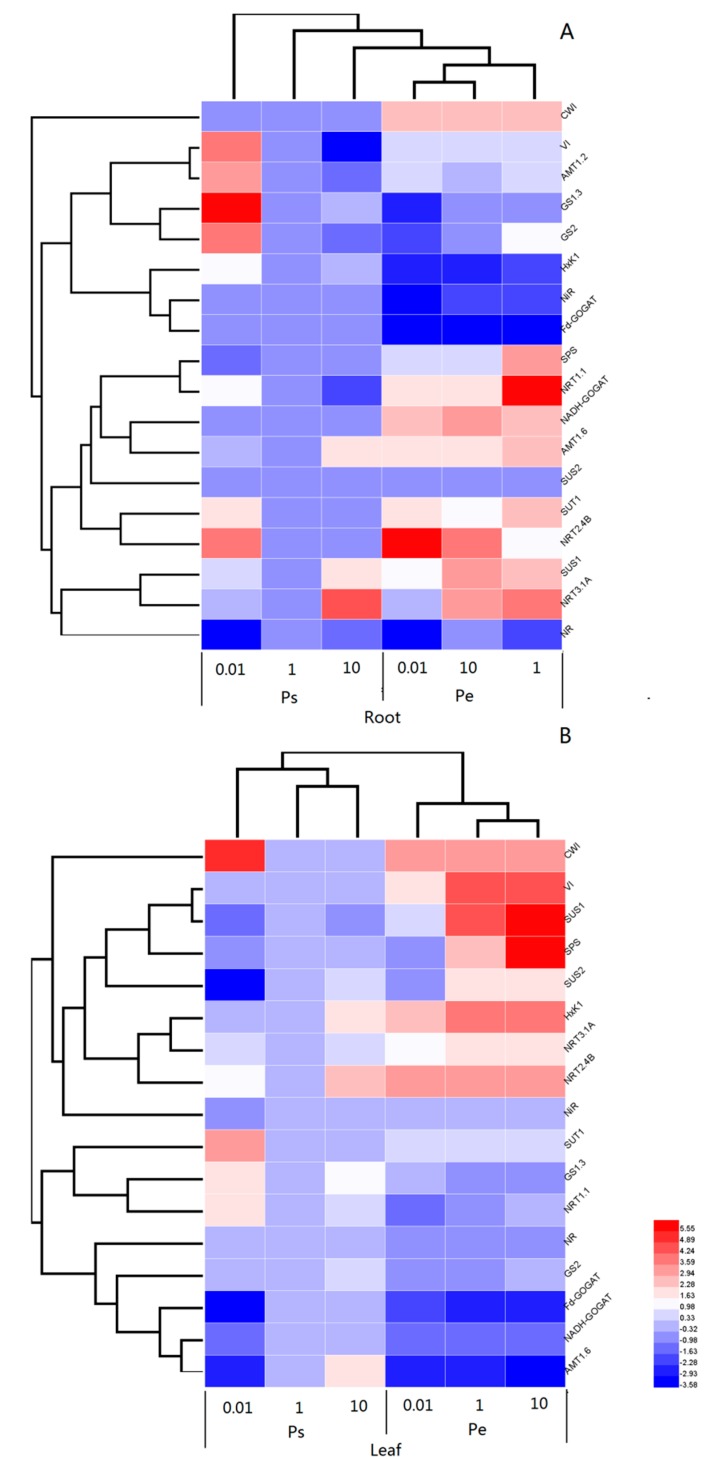
Cluster analysis of transcriptional fold-changes of key genes involved in N uptake and assimilation in roots (**A**) and leaves (**B**) of *P. simonii* (Ps) and *P. euramericana* (Pe) under 0.01, 1, and 10 mM NH_4_NO_3_. The colour scale indicates fold-changes of mRNAs. For each gene, the expression levels in roots or leaves of Pp exposed to 1 mM NH_4_NO_3_ were defined as 1, and the corresponding fold-changes under 0.01 and 10 mM NH_4_NO_3_ were calculated.

In the roots, Pe was more responsive to the N supply than Ps (Figure 9A). The mRNA levels of *CWI*, *NiR*, *Fd*-*GOGAT*, and *SUS2* in the roots were fairly consistent at all three N supply levels in both poplars (Figure 9A). The transcript levels of *VI*, *AMT1.2*, *GS1.3*, *GS2*, *HxK*, *NRT1.1*, *SUT1*, and *NRT2.4B* were upregulated in the Ps roots when exposed to low N supply, but the responses of these genes to the N supply varied in Pe (Figure 9A). For instance, the transcript levels of *VI* and *HxK* in the Pe roots were not altered in response to the N level (Figure 9A), but the mRNA levels of *NRT2.4B* were increased in the Pe roots with low N supply (Figure 9A). However, *GS1.3*, *GS2*, and *NRT1.1* were suppressed in the Pe roots in response to low N supply (Figure 9A). The transcript levels of *SPS*, *SUS1*, *NRT3.1A*, and *NR* increased with N supply in the toots of both Ps and Pe (Figure 9A).

In leaves, increased transcription of *CWI*, *SUT1*, *GS1.3*, and *NRT1.1* were detected in Ps in response to N deficiency, but no such effects were found in Pe (Figure 9B). The mRNA levels of *SUS1*, *SUS2*, *HxK*, *NRT2.4B*, *NiR*, *Fd*-*GOGAT*, *NADH*-*GOGAT*, and *AMT1.6* were decreased in the leaves of Ps in response to low N supply, but in the leaves of Pe, the transcript levels of these genes showed differing responses to N deficiency (Figure 9B). The transcript levels of *SUS1*, *SUS2* and *HxK* showed a regulatory pattern in the leaves of Pe similar to that in the leaves of Ps (Figure 9B). However, the mRNA levels of *NRT2.4B*, *NiR*, *Fd*-*GOGAT*, *NADH*-*GOGAT*, and *AMT1.6* in the leaves of Pe were largely unchanged in response to the N supply (Figure 9B). 

## 3. Discussion

### 3.1. The Slow-Growing Ps Exhibits Enhanced Resistance to N Deficiency

Root morphological traits are strongly affected by the soil N availability [23,25]. Some studies have found that high N supply promotes root development and results in a greater root biomass [26]. However, conflicting results have also been reported, with the nitrogen supply having little or no effect on root growth [27]. In addition, the N supply leads to different effects on root growth; N deficiency induced longer roots, greater surface area and greater biomass in the slow-growing Ps (Table 1). Consistent with root growth, low N supply had only minor effects on the photosynthetic pigments (*A*, *gs*, *E*).

Metabolites of C generated from photosynthesis are major building blocks for growth and contribute to differences in acclimation to N limitation in poplars. In Ps, under a limiting N supply, photosynthate allocation was prioritized to root growth. Indeed, in Ps, root sucrose concentrations were higher with low N supply than with medium or high N supply. Low N supply might have caused greater export of photosynthate from source to sink tissues in Ps. Consistent with this hypothesis, Ps also showed a lower foliar sucrose content with low N supply. A higher total C concentration induced SPS and HxK activity in the leaves in Ps with abundant N supply, which indicated that C metabolism is stimulated by a sufficient N supply (Figure 3D). Indeed, it has also been shown in other plants that an increase in protein biosynthesis results in a greater demand for C skeletons [28,29]. However, the activities of HxK, CWI, and VI in the Ps roots were induced with low N supply, suggesting accelerated glycogen metabolism and respiration in the Ps roots in response to N deficiency. The sucrose in the roots is transported from the source tissue, hydrolysed and phosphorylated to glucose-6-phosphate, which is the key substrate for glycolysis and respiration [30]. These results were consistent with the greater root development seen in Ps with low N supply because more energy was required to support root growth.

Fractionation of nitrogen isotopes between a plant and its environment occurs during uptake and assimilation of inorganic nitrogen [20]. δ^15^N at natural abundance levels acts as a tracer, and significant discrimination is positive in most biological systems. For instance, *Populus* × *euramericana* and *Populus* × *cathayana* display increased δ^15^N under drought condition [31]. In other plants, δ^15^N is also increased by an N deficiency [32,33]. Low N supply leads to higher δ^15^N and decreases the total N concentration, suggesting that δ^15^N is enriched in N-deficient poplars. Under N deficiency, plants retard their N uptake and assimilation processes and are forced to utilize ^15^N to meet N demands; thus, δ^15^N is enriched in the tissues [34,35]. The higher δ^15^N under low N supply compared with those under medium and high N supply is probably associated with slowing of the N metabolism, leading to less depletion of δ^15^N under low N availability (Figure 6B,D). These results were consistent with the decreased net NH_4_^+^ and NO_3_^−^ flux, the total N concentration and the NR activity. However, low N supply induced the activities of enzymes involved in NH_4_^+^ assimilation (i.e., GS, GOGAT, and GDH) in Ps leaves. The slow-growing Ps is widespread on the Loess plateau. The soil in this area is alkaline and low in N. NH_4_^+^ can stimulate root development and Ps may have evolved a mechanism to utilize limiting NH_4_^+^ even in a nutrient deficient soil [23,36]. These results are consistent with a flourishing root system in Ps in the presence of low N.

The transcriptional regulation of key genes involved in C/N metabolism is crucial for herbaceous plants in response to a complex and volatile N supply [37,38]. However, little is known about transcriptional regulation in trees at deficient and sufficient N levels. Previous studies have detected higher expression, downregulation, or stable transcript levels of key genes involved in C/N metabolism in various poplar species in response to low and/or sufficient N levels [28,39]. The transcriptional downregulation of *SUS1* and *SPS* in response to N deficiency indicates that C metabolism is inhibited due to a limited N supply. However, the transcript levels of *SUT*, *HxK* and *VI* in roots of Ps were induced by N deficiency, and this is consistent with the higher activities of carbohydrate catabolism and greater root growth at low N levels.

In plants, nitrate transporters are mostly encoded by two gene families, namely *NRT1* and *NRT2*. *NRT1* genes encode members of low affinity transport system (LATS) and *NRT2* genes encode high affinity transporters (HATS). In *populus*, the major roles in LATS and HATS were played by *NRT1.1* and *NRT2.4*, respectively. In addition, the accessory protein *NRT3* is required for a functioning NO_3_^–^ transport [40]. The transcript levels of *AMT1.2* and *NRT2.4B* were increased by low N levels, suggesting that poplar roots increase the mobilization of NH_4_^+^ and NO_3_^−^ in response to low N availability. Similarly, the induction of the transcription of *AMT1.2* in roots has also been found in *P. tremula × tremuloides*, *P. tremula × alba* and rice (*Oryza sativa*) [41,42]. In contrast to the induction of several *AMTs* and *NRTs*, reduced transcript levels of most genes involved in N assimilation (e.g., *NR*, *NiR*, *GS2*, and *GOGAT*) indicate that N assimilation is retarded under N deficiency. These results were consistent with the activities of the enzymes involved in C/N metabolism.

### 3.2. The Fast Growth of Pe Relies on Sufficient N Supply

In our study, the root length and surface area were higher in Pe than in Ps with sufficient N supply, but were similar between the two poplars with low N supply (Table 1). In addition, Pe had greater biomass than Ps with a sufficient N supply. These results indicate that the fast-growing Pe was more sensitive to the N supply than the slow-growing Ps and that Pe had a greater capacity for nitrogen acquisition and assimilation. This characteristic of the fast-growing Pe may be critical for supporting its rapid metabolism and growth [43]. With rapid metabolism and growth, Pe may develop a flourishing root system that results in greater nutrient absorption to satisfy metabolic consumption.

Photosynthesis is the primary limiting factor of plant productivity. N is a constituent of the photosynthetic machinery, and N-containing compounds play an essential role in CO_2_ fixation [44]. Plant productivity depends on the plant and the metabolic expenditure [45]. The chlorophyll content and the net photosynthetic rate in Ps were higher than in Pe at low N, suggesting that the fast-growing Pe has low resistance to N deficiency. However, the total carbohydrates and the net photosynthetic rate were higher in Pe than in Ps, indicating that Pe has a greater carbon assimilation capacity than does Ps (Figure S1). Actually, most fructose and glucose content measurements in the roots and leaves in Pe were similar to, or higher than, those in Ps at all three N levels. However, with low N supply, the root sucrose content was lower in Pe than in Ps. Sucrose is the form in which carbohydrates are transported from the source to the sink tissues and reflects the C export status [10]. Compared with the fast-growing Pe, Ps may have evolved an adaptive C transport strategy in the presence of low N. These results indicate that the fast-growing Pe was superior to the slow-growing Ps in utilizing N to stimulate growth and produce biomass. Additionally, with a sufficient N supply, the activities of enzyme involved in C metabolism (i.e., SPS, SUS, HxK, CWI, and VI activities) were also higher in Pe than in Ps, indicating acceleration of the physiological processes of C metabolism in Pe compared with Ps (Figure 3). 

N physiological process is critical for plant growth and may differ between species. Indeed, the NH_4_^+^, NO_3_^−^, and total N concentrations were generally higher in the roots and leaves of Pe than in Ps. Consistent with these results, the net flux of NH_4_^+^ and NO_3_^−^ in the Pe roots was also higher than in the Ps roots, reflecting the ability of the fast-growing Pe to acquire NH_4_^+^ and NO_3_^−^ more rapidly. However, δ^15^N in the Pe roots was lower than in Ps. N assimilation does not favour ^15^N enrichment and results in the depletion of ^15^N in the plant dry matter compared with the heavier N isotope in the soil [35,46]. Previous studies showed that slow-growing poplars accumulate N via a decrease in the nitrogen-use efficiency, resulting in greater N storage [5,47]. These results were consistent with lower N uptake (i.e., the net flux and concentration of NH_4_^+^ and NO_3_^−^), and lower total N concentration and assimilation (i.e., the NR, GS, GDH activity) in Ps compared with Pe. 

Associated with the different morphological and physiological responses to the N supply, different transcriptional regulation of the genes and post-transcriptional/translational mechanisms involved in C/N metabolism are also expected in the two poplar species [48]. In our study, the regulation of most key genes was more responsive to N addition in Pe than in Ps. Many studies have also reported differences among poplar species in gene expression responses to high temperature, drought stress and nutrient availability [3,23,49]. For example, N fertilization leads to the acceleration of N physiological processes via *AMTs* and *NRTs* in *Populus popularis*, but not in *Populus alba × Populus glandulosa* [8]. These results suggest that fast-growing poplars may utilize N nutrition more efficiently than slow-growing poplars. The flourishing root system, higher δ^15^N, accelerated C export, lower N uptake and assimilation and less sensitive transcriptional regulation in the slow-growing Ps compared to the fast-growing Pe contribute to two species’ different patterns of the physiological and molecular responses to deficient and sufficient N supply.

## 4. Materials and Methods

### 4.1. Plant Growth Conditions and Treatments

Cuttings of *P. simonii* and *P. euramericana* were made from one-year-old stems, approximately 15 cm in length and 2 cm in diameter, and were then germinated and grown in fine sand for four weeks. One strong bud was left on each sapling at the beginning of sprouting. Saplings that evidenced similar growth were subsequently cultivated hydroponically. For each species, 36 plants were divided into three groups. The plants in the three groups of each species were treated with modified Hoagland’s solution (10 μM EDTA·FeNa, 0.5 μM H_2_MoO_4_, 30 μM H_3_BO_3_, 1 μM CuSO_4_·5H_2_O, 1 μM ZnSO_4_·7H_2_O, 5 μM MnSO_4_·H_2_O, 1 mM MgSO_4_·7H_2_O, 1 mM KH_2_PO_4_, 1 mM Na_2_SO_4_, 1 mM CaCl_2_) containing NH_4_NO_3_ at 0.01, 1, or 10 mM, representing low, medium and high N levels, respectively. The plants were cultivated in a greenhouse (natural light; day/night temperature: 25/20 °C; relative humidity: 75%). During the experiment, the volume of hydroponics containers was sufficient (2 m × 1 m × 0.5 m), and the aerated nutrient solutions were renewed every other day to ensure that plant uptake did not alter solution concentration; the experimental duration was four weeks. In each group, six plants were used for the determination of growth parameters and the remaining six plants were used for measurements of physiological and molecular characteristics.

### 4.2. Photosynthetic Characteristics and Root Development

The photosynthetic characteristics (i.e., the net photosynthetic rate (*A*), stomatal conductance (*g_s_*) and transpiration rate (*E*)) in three mature leaves with a leaf plastochron index = 8–10 were measured. The net photosynthetic rate was measured from 9:00 to 11:00 h in the greenhouse using a portable photosynthesis system (Li-Cor-6400; Li-Cor, Inc., Lincoln, NE, USA) with an attached LED light source (1000 μmol photon m^−2^ s^−1^). The CO_2_ concentration in the chambers was 400 μmol·mol^−1^, and the air flow was 500 mmol s^−1^. The chlorophyll content was measured with a portable chlorophyll meter (SPAD 502 Meter, Minolta Corporation, Tokyo, Japan).

After the gas exchange measurement, the plants were harvested and separated into roots and shoots. The root system was washed with the appropriate modified Hoagland’s solution and wrapped in laboratory tissue paper to remove the water from the root surface. The fresh weight of the roots and shoots was recorded. Part of the root system (approximately 2 g) was excised from each plant and then scanned and analysed by using TWAIN PRO (32 bit) and a WinRHIZO root analyser system (WinRHIZO version 2007b, Regent Instruments Canada, Montreal, QC, Canada). Subsequently, the samples were wrapped in tinfoil, flash-frozen in liquid nitrogen and stored at −80 °C.

### 4.3. Determination of Contents of Sucrose, Fructose, Glucose, and Total Carbon

Total soluble carbohydrates were extracted and analysed according to the method of Zhao et al. [10]. with minor modification. Briefly, 200 mg of frozen powder was homogenized with 1 mL of protein extraction buffer (50 mM Na-acetate, pH 5; 10 mM NaHSO_3_; 0.02% (*w*/*v*) Na-azide; and 0.1% (*w*/*v*) Polyclar AT) for 15 min at 95 °C. After centrifugation (1000× *g*, 5 min), the supernatant was centrifuged again at 10,000× *g* for 5 min. Sucrose, fructose, and glucose concentrations were quantified by using a high- performance anion-exchange chromatography with pulsed amperometric detection (HPAEC-PAD).

### 4.4. Determination of Activities of Enzymes Involved in C Metabolism in Roots and Leaves

Sucrose phosphate synthase activity (SPS, EC 2.4.1.14), sucrose synthase activity (SUS, EC 2.4.1.13), and hexokinase activity (HxK, EC 2.7.1.1) in roots and leaves were measured according to the instructions provided by the manufacturer of the assay kit (JCbio, Nanjing, China). 

For SPS, approximately 1 g of frozen material was ground to a fine powder in an icebath with 5 mL of 4-(2-hydroxyethyl)-1-piperazineethanesulfonic acid-NaOH buffer (50 mM, pH 7.5) containing 50 mM MgCl_2_, 2 mM EDTA, 0.2% (*w*/*v*) bovine serum albumin, and 2% polyvinyl pyrrolidone (PVP). After centrifugation (10,000× *g*, 10 min), 50 μL of supernatant was mixed with 50 μL of HEPES-NaOH buffer, 20 μL of 50 mM MgCl_2_, 20 μL of 100 mM uridine diphosphoglucose (UDPG), and 20 μL of 100 mM fructose. The mixture was incubated at 30 °C for 30 min, and the reaction was stopped by the addition of 200 μL of 2 mM NaOH and boiling for 10 min. The solution was then cooled to room temperature. Next, 1.5 mL of 30% HCl and 0.5 mL of 0.1% resorcin were added and mixed thoroughly. Then, the mixture was incubated at 80 °C for 10 min. The solution was cooled to room temperature again and light absorption was measured spectrophotometrically at 480 nm. The sucrose concentration was calculated from a standard curve of known sucrose concentrations.

For SUS, 0.5 g of the frozen material was ground to a fine powder in 5 mL of ice cold extraction buffer containing 100 mM Tris-HCl (pH 7.0), 5 mM MgCl_2_, 2 mM EDTA-Na_2_, 2% glycol, 0.2% bovine serum protein (BSP), 2% PVP, and 5 mM dithiothreitol (DTT). The extract was centrifuged at 10,000× *g* for 20 min at 2 °C. Then, 3 mL of the supernatant was dialyzed overnight at 4 °C. The dialysate (25 mM Tris-HCl (pH 7.0), 2.5 mM MgCl_2_, 1 mM EDTA-Na_2_, 1% glycol, 1 mM DTT) was refreshed every 4 h. Next, 0.1 mL of enzyme extract, 0.1 mL of 10 mM UDPG, 0.4 mL H_2_O, and 0.4 mL of reaction solution (100 mM Tris-HCl (pH 7.0), 10 mM fructose, 2 mM EDTA-Na_2_, 5 mM magnesium acetate, 5 mM DTT) were incubated at 30 °C for 10 min. The reaction was stopped by boiling for 3 min. Then, 0.1 mL of 2 mM NaOH was added to the mixture, followed by boiling for 10 min. After cooling, 3.5 mL of 30% HCl and 1 mL of 0.1% resorcin were added, and the mixture was incubated at 80 °C for 10 min. The mixture was then cooled to room temperature again and the sucrose concentration was measured at 480 nm using the standard sucrose curve.

For HxK, crude extracts were made by grinding frozen material (0.5 g) to a fine powder in liquid nitrogen. The fine powder was homogenized with 5 mL of ice- cold extraction buffer containing 20 mM KH_2_PO_4_ (pH 7.5), 0.5 mM EDTA, and 5 mM DTT. After centrifugation (12,000× *g*, 20 min), the crude extract was desalted with Sepahdex G25 columns. HxK activity was quantified spectrophotometrically at 340 nm in 1 mL of reaction solution containing 50 mM Tris-HCl (pH 8.5), 10 mM MgCl_2_, 110 mM glucose, 0.2 mM NADH, 0.5 mM ATP, two units of glucose-6-phosphate dehydrogenase (EC 1.1.1.49), and 33 μL of crude extract.

The activities of cell wall and vacuolar invertase (EC 3.2.1.26) were analysed according to the methods described by Link et al. [50]. Briefly, approximately 1 g of tissue was ground to a fine powder in liquid nitrogen and homogenized in 2 mL of extraction buffer (30 mM 3-(*N-*morpholino) propanesulfonic acid, 250 mM sorbitol, 10 mM MgCl_2_, 10 mM KCl, and 1 mM Phenylmethanesulfonyl fluoride, pH 6). The mixture was centrifuged at 3500× *g* for 10 min. The pellet was washed twice with extraction buffer and resuspended in 1 mL of assay buffer (20 mM triethanol amine, 7 mM citric acid, and 1 mM PMSF, pH 4.6), and used for the determination of cell wall invertase activity. The supernatant was mixed with 18 mL of ConA buffer (500 mM sodium acetate, 10 mM CaCl_2_, 10 mM MgCl_2_, 10 mM MnCl_2_, and 1 mM PMSF, pH 6.3), and 20 μL of concanavalin A (ConA)-sepharose and then agitated in an ice bath for 1 h. The solution was centrifuged at 3000× *g* for 5 min and then washed with 100 mL of ConA buffer. The bound protein fraction was then eluted with 100 μL of elution buffer (50 mM sodium acetate, 1 mM CaCl_2_, 1 mM MgCl_2_, 1 mM MnCl_2_, and 0.1 mM PMSF, pH 6.3, 10% (*w*/*v*) methyl α-d-glucopyranoside). The solution was then measured spectrophotometrically for vacuolar invertase activity.

### 4.5. Determination of Net Fluxes of NH_4_^+^, NO_3_^−^, and H^+^

The net fluxes of NH_4_^+^, NO_3_^−^, and H^+^ in the roots were measured by using a non-invasive micro-test technique (NMT, system BIO-IM, Amherst, MA, USA) as described in our previous study [23]. Briefly, silanized glass micropipettes were first filled with a backfilling solution (100 mM NH_4_Cl for the NH_4_^+^ electrode; 10 mM KNO_3_ for the NO_3_^−^ electrode; and 15 mM NaCl and 40 mM KH_2_PO_4_ for H^+^ electrode). Then, 50 μm columns of selective liquid ion-exchange cocktails (NH_4_^+^ LIX, #09879, Sigma, Amherst, MA, USA; NO_3_^−^ LIX, #72549, Sigma; H^+^, #95293, Sigma) were front-filled. An Ag/AgCl wire electrode holder (XY-DJGD, Younger USA, LLC, Amherst, MA, USA) was inserted into the back of the electrode. The microelectrodes were then calibrated and used for measurement. A fine root was equilibrated for 20 min in measuring solution (0.1 mM KCl, 0.1 mM CaCl_2_, pH 5.5) with 0.01, 1, or 10 mM NH_4_NO_3_ according to the N treatment of the selected root. Six white fine roots were selected from the root system for each treatment. 

Ion gradients near the root surface (approximately 5 μm above the root surface) were measured by moving an ion-selective microelectrode between two positions that were 30 μm apart and perpendicular to the root axis. The recordings were performed every 6 s for an average of 10 min at each measurement point.

### 4.6. Determination of Contents of NH_4_^+^, NO_3_^−^, NO_2_^−^, δ^15^N, and Total N

NH_4_^+^ content in the roots and leaves was spectrophotometrically analysed as previously described [14]. Approximately 100 mg of the fine powder was homogenized in extraction solution containing 1 mL of 100 mM HCl, and 500 μL of chloroform. The mixture was rotated for 15 min at 4 °C and then centrifuged (10,000× *g*, 4 °C, 10 min). The supernatant was transferred to a 2 mL tube, and 50 mg of activated charcoal was added and mixed thoroughly. After centrifugation (12,000× *g*, 4 °C, 5 min), the NH_4_^+^ content in the supernatant was measured at 620 nm.

NO_3_^−^ content was quantified based on the method of Patterson et al. [51]. First, 100 mg of the frozen powder was extracted in 1 mL of deionized water (45 °C, 1 h). The solution was then centrifuged (5000× *g*, 20 °C, 15 min) and 0.2 mL of the supernatant was transferred to a 2 mL tube containing 0.8 mL of 5% (*w*/*v*) salicylic acid in concentrated H_2_SO_4_ and incubated for 20 min at 25 °C. Then, 2 M NaOH was added to raise the pH to above 12. The NO_3_^−^ content in the solution was measured spectrophotometrically at 410 nm.

NO_2_^−^ content was assayed as described by Ogawa et al. [52]. Approximately 100 mg of the fine powder was homogenized in extraction buffer containing 50 mM TRIS-HCl (pH 7.9), 5 mM cysteine, and 2 mM EDTA and centrifuged (10,000× *g*, 20 °C, 20 min). Then, 500 μL of the supernatant, 250 μL of 1% sulfanilamide and 250 μL of 0.02% N-(1-naphthyl) ethylenediamine dihydrochloride in 3.0 M HCl were combined and mixed well. The NO_2_^−^ content was spectrophotometrically determined at 540 nm.

*δ*^15^N and total N concentration in roots and leaves were analyzed as proposed by Evans et al. [21]. The samples were dried in an oven at 80 °C. The isotopic ratio and total N concentration were analysed by using an elemental analyser (NA 1110, CE Instruments, Rodano, Italy) coupled to a GVI IsoPrime isotope ratio mass spectrometer (IRMS). Stable N isotope composition was calculated as:

*δ*^15^N = (R_san_ − R_sdn_)/R_sdn_ × 1000

where R_san_ and R_sdn_ are the ratios of ^15^N to ^14^N in the sample and the standard, respectively. The reference standard was N_2_ in air.

### 4.7. Determination of Activities of Enzymes Involved in N Metabolism in Roots and Leaves

NR activity (EC 1.7.99.4) in the roots and leaves was determined as previously described [53]. Fine powder (1 g) was homogenized in 4 mL of ice cold extraction buffer (25 mM phosphate buffer (K_2_HPO_4_ and KH_2_PO_4_, pH 7.5), 5 mM cysteine and 5 mM EDTA-Na_2_). After centrifugation (4000× *g*, 4 °C, 15 min), 0.4 mL of supernatant and 1.6 mL of the assay solution (1.2 mL of 0.1 M KNO_3_-phosphate buffer and 0.4 mL of 2.0 mg mL^−1^ NADH) were mixed well and incubated at 25 °C for 30 min). The reaction was terminated by adding 1 mL of 1% (*w*/*v*) sulfanilamide in 3 N HCl and 0.02% N-naphthylethylenediamine in water. After incubation for 15 min, the solution was then centrifuged for 10 min at 4000× *g* followed by spectrophotometric measurement of the supernatant at 540 nm and determination of the NO_2_^−^ concentration by using a standard curve of known NO_2_^−^ concentrations.

The activity of NiR (EC 1.7.2.1) was measured according to the methods described by Seith et al. [54]. Briefly, 0.5 g of the fine powder was extracted in the extraction buffer consisting of 25 mM phosphate buffer (K_2_HPO_4_ and KH_2_PO_4_, pH 7.5), 5 mM cysteine, and 5 mM EDTA-Na_2_. The crude extract was mixed with 0.1 M potassium phosphate buffer (pH 6.8), 0.4 mM NaNO_2_, 2.3 mM methyl viologen, and 4.3 mM sodium dithionite in 100 mM NaHCO_3_. The mixture was incubated for 30 min at 27 °C, and the reaction was stopped by boiling for 5 min. The concentration of NO_2_^−^ remaining in the reaction mixture was determined at 540 nm; one unit of NiR activity was defined as 1 mmol of NO_2_^−^ reduced mg^−1^ protein h^−1^.

The activity of GS (EC 6.3.1.2) was analysed spectrophotometrically, as proposed by Wang et al. [55]. Briefly, frozen fine powder (approximately 1 g) was homogenized in ice cold 50 mM Tris-HCl extraction buffer (pH 8.0) containing 2 mM MgCl_2_, 2 mM DTT, and 0.4 M sucrose. The solution was centrifuged (15,000× *g*, 4 °C, 20 min) and the supernatant was mixed with the assay solution (0.35 mL of 40 mM ATP and 0.8 mL of 0.1 M Tris-HCl buffer (pH 7.4) with 20 mM Na-glutamate, 80 mM MgSO_4_, 20 mM cysteine, 2 mM EGTA, and 80 mM NH_2_OH). After incubation for 30 min at 37 °C, the stop reagent (0.37 M FeCl_3_, 0.2 M trichloroacetic acid, 0.6 M HCl) was added. After centrifugation (5000× *g*, 4 °C, 15 min), the supernatant was spectrophotometrically measured at 540 nm; one unit of GS activity was defined as 1μmol of γ-glutamyl hydroxamate formed per min.

GOGAT (EC 1.4.7.1) and GDH (EC 1.4.1.2) activities were determined as suggested by Lin and Kao [56]. Briefly, 0.5 g of the fine powder was extracted in 0.2 M sodium phosphate buffer (pH 7.5) containing 2 mM EDTA, 50 mM KCl, 0.1% (*v*/*v*) mercaptoethanol and 0.5% (*v*/*v*) Triton X 100. After centrifugation (6000× *g*, 4 °C, 15 min), the supernatant was used for measurement of GOGAT and GDH activities. For GOGAT, 0.3 mL of enzyme extract was mixed with 0.2 mL of 20 mM l-glutamine, 50 μL of 0.1 M 2-oxoglutarate, 100 μL of 10 mM KCI, and 0.1 mL of 3 mM NADH. The absorbance was measured at 340 nm for 5 min. For GDH, 0.15 mL of 0.1 M 2-oxoglutarate, 0.15 mL of 1 M NH_4_Cl, 0.1 mL of 3 mM NADH, and 0.6 mL of 0.2 M Tris-HCl buffer (pH 8.0) were mixed and 0.5 mL of the supernatant was added. The decrease in absorbance at 340 nm was measured.

### 4.8. RNA Extraction and Analysis of the Transcript Levels of Key Genes Involved in C/N Metabolism

Total RNA was extracted and purified using the Omega reagent (R6827, Omega Bio-Tek, Norcross, GA, USA) according to the manufacturer’s instructions. The OD_260_/OD_280_ and electrophoresis were used to assess RNA quality. cDNA was prepared from DNase-treated RNA using a PrimeScript RT reagent kit (DRR037S, Takara, Dalian, China). The SYBR Green (Roche, Basel, Switzerland) method was used to perform qRT-PCR in a Roche real-time PCR system (LightCycler 96, Roche, Basel, Switzerland). The poplar actin 2/7 gene was used to standardize the expression level of all genes tested. A list of primers and their efficiencies is given in Table S1.

### 4.9. Statistical Analysis

The net flux results were calculated by using MageFlux version 1.0 attached to the NMT system (available online: http://cn.xuyue.net/). Statistical analyses were performed using SPSS (SPSS Inc., Chicago, IL, USA). The data were analyzed using a two-way ANOVA after a normality test. A post-hoc analysis was performed using LSD method. Differences between the means were assessed on the basis of the least significant difference (*p* = 0.05). The cluster analysis of gene expression was mapped with the HemI software (Heatmap Illustrator, version 1.0, Wuhan, China).

## 5. Conclusions

Taken together, compared with the slow-growing Ps, the fast-growing Pe showed greater root development, C/N uptake and assimilation capacity, and more responsive transcriptional regulation with an increasing N supply. With N deficiency, a lower photosynthetic rate is consistent with lower N acquisition, lower NR, SPS, and HxK activities and the downregulation of most genes involved in C/N metabolism in both species. Moreover, N deficiency induced greater root growth, greater C export from the leaves to the roots, and greater NH_4_^+^ assimilation (i.e., GS, GOGAT, GDH activity, and the genes that encode them) in Ps, and these effects were not detected in the fast-growing Pe. Compared with the fast-growing Pe, the slow-growing Ps showed a greater resistance to N deficiency, as indicated by the transport of photosynthate to the roots, which stimulated root development for survival, and this was associated with their native environment. These morphological, physiological, and transcriptional regulatory strategies indicate that poplar species can differentially manage C/N metabolism and photosynthate allocation at various N levels. This is important when selecting poplar species for different soil conditions.

## Figures and Tables

**Table 1:** Growth and photosynthetic characteristics of slow growing *P. simonii* (Ps) and fast growing *Populus × euramericana* (Pe) plants exposed to 0.01, 1, or 10 mM NH_4_NO_3_ (N).

Species	N Supply (mM)	Root Biomass (mg DW)	Total Fine Root Length (m)	Total Fine Root Surface Area (cm^2^)	Total Root Volume(cm^3^)	SPAD Values	*A* (μmol CO_2_ m^−2^ s^−1^)	*g_s_* (mol H_2_O m^−2^ s^−1^)	*E* (mmol H_2_O m^−2^ s^−1^)
Ps	0.01	5.74 ± 0.79 ^bc^	13.62 ± 1.60 ^c^	57.55 ± 5.62 ^c^	12.72 ± 1.38 ^bc^	22.90 ± 3.44 ^b^	11.86 ± 1.03 ^a^	0.22 ± 0.02 ^ab^	4.85 ± 0.37 ^a^
	1	3.93 ± 0.53 ^d^	11.77 ± 1.58 ^cd^	40.25 ± 3.53 ^d^	11.73 ± 1.13 ^c^	28.82 ± 2.74 ^a^	9.75 ± 0.61 ^b^	0.26 ± 0.02 ^a^	4.97 ± 0.46 ^a^
	10	2.73 ± 0.35 ^e^	9.53 ± 1.18 ^d^	48.21 ± 3.67 ^cd^	9.73 ± 0.98 ^c^	31.23 ± 2.88 ^a^	10.23 ± 0.82 ^ab^	0.22 ± 0.05 ^ab^	4.93 ± 0.41 ^a^
Pe	0.01	4.93 ± 0.58 ^c^	15.14 ± 1.60 ^bc^	98.66 ± 11.45 ^b^	12.72 ± 2.21 ^bc^	14.58 ± 1.11 ^c^	6.25 ± 1.03 ^c^	0.09 ± 0.02 ^d^	2.67 ± 0.21 ^c^
	1	7.37 ± 0.54 ^a^	22.95 ± 1.59 ^a^	130.24 ± 12.89 ^a^	17.92 ± 3.05 ^a^	29.77 ± 1.87 ^a^	9.45 ± 0.71 ^b^	0.15 ± 0.02 ^c^	3.26 ± 0.32 ^bc^
	10	6.18 ± 0.49 ^b^	17.75 ± 4.05 ^b^	109.05 ± 12.47 ^b^	15.85 ± 1.30 ^ab^	31.22 ± 2.75 ^a^	11.09 ± 1.56 ^ab^	0.18 ± 0.03 ^bc^	3.69 ± 0.20 ^b^
*p*-values	Species	***	***	***	***	**	***	***	***
	N	***	***	ns	*	***	**	***	**
	Species × N	***	***	***	***	***	***	**	**

Data indicate mean ± SE (*n* = 6). Different letters in the same column indicate significant difference (*p* < 0.05). *p*-Values of the ANOVA of drought, nitrogen supply, and their interaction are indicated. * *p* < 0.05; ** *p* < 0.01; *** *p* < 0.001; ns, not significant. Net photosynthetic rates (*A*), stomatal conductance (*gs*), and transpiration rates (*E*).

## Supplementary Materials

Supplementary materials can be found at http://www.mdpi.com/1422-0067/19/8/2302/s1.

## Abbreviations

AMTs	Ammonium transporters
CWI	Cell wall invertase
GDH	Glutamate dehydrogenase
GOGAT	Glutamate synthase
GS	Glutamine synthetase
HxKs	Hexokinases
NiR	Nitrite reductase
NR	Nitrate reductase
NRTs	Nitrate transporters
SPP	Sucrose-6-phosphate phosphatase
SPS	Sucrose phosphate synthase
SUS	Sucrose synthase
SUT	Sucrose transporter
UDP-G	Uridine diphosphate glucose
VI	Vacuolar invertase
