# Supplementary material for: Distinct Carbon and Nitrogen Metabolism of Two Contrasting Poplar Species in Response to Different N Supply Levels
Source: Int J Mol Sci. 2018 Aug 6;19(8):2302. doi: 10.3390/ijms19082302 (PMC6121361; doi:10.3390/ijms19082302)
Supplement: Supplementary file 1 [file ijms-19-02302-s001.pdf]

Supplementary Materials: Distinct Carbon and Nitrogen Metabolism of Two Contrasting Poplar Species in Response to Different N Supply Levels

Sen Meng, Shu Wang , Jine Quan, Wanlong Su, Conglong Lian, Dongli Wang, Xinli Xia and Weilun Yin

Table S1. Primers used for qRT-PCR.

Gene Model	Gene Name	Closest AGI	Primer-Forward (5′-3′)	Primer-Reverse (5′-3′)	PCR Efficiency (%)
Potri.019G023600	*AMT1;2*	AT1G64780	CCTCCAATGGGTCATCATATTG	GTCATTGGAATGTGATTGATGTATG	100
Potri.009G045200	*AMT1;6*	AT4G13510	CACTCCCGCCTCACCTGAAT	TGGGCTCAACGTCTTGGCTCT	94.5
Potri.004G179400	*NRT1;1*	AT1G66980	GCCTTCCTCAGCGACTCTTACT	GAATGTTCATCACCACAGCCTCTC	101
Potri.009G008500	*NRT2;4a*	AT5G60770	TGCTGTCACCGCTATGATTCTCT	ATGATAGACCTGCCGCTGTGG	97
Potri.012G089300	*NRT3;1a*	AT5G50200	CCTTGCTATGGAATTGTGCTCTTCT	GATGGTCTTGTAGGTCGAGTCAGT	97.5
Potri.005G172400	*NR*	AT1G37130	ATCATCGGATCGGAGAGTTGG	GACGGTGCTAGTTGGCGTATAG	99
Potri.004G140800	*NiR*	AT5G37600	ACAAGTTGCCGATATTGGGTTCAT	CCTCTATCACCCGTCGTAGTCCTG	105
Potri.016G036900	*Fd-GOGAT*	AT5G04140	AACCCAAAGGCATCAGACTCAG	AGTAAAGCAGGTCCATCCCAAG	102
Potri.012G011700	*NADH-GOGAT*	AT5G53460	GGTGTTGTGGATATTCCTCCTG-	TCAGATGCGGCGACAACCC	98
Potri.012G043900	*GS1;3*	AT5G37600	TGGAAACCATAAGAGATCACCACC	GAAGAGGCAATTCTTGTACCAAG	101
Potri.010G029100	*GS2*	AT5G35630	GGAGCATCACTTGGATCTAGATGG	CAAAACCCAAGAGTAAAAAGGTCC	104
Potri.006G227400	*CWI*	AT3G13790	TCGTAGACATGGATCCTCGC	TGCTTGTGAATTGCCAGCTT	97
Potri.007G108300	*VI*	AT5G64620	CAGTTCAAGATCTGGCCACA	GCAAGCTCGGATGGATAAGC	102
Potri.001G254800	*HxK*	AT1G50460	CCATTCCCCAGCACTTGATG	GAAGGCTCGGAACCACTTTC	104
Potri.006G064300	*SPS*	AT1G04920	ACTCACTTGGCCGGGATAAA	ACTCCTCTGCCTCTATCCGA	101
Potri.006G136700	*SUS1*	AT5G49190	CTCGCCCAACTCTTTCGAAG	TGTGACAGTGCACCTTGAGA	104
Potri.018G063500	*SUS2*	AT5G49190	CAACATTGTATCCCCTGGCG	AGGGCTGTAAAGAAGCTCGT	99
Potri.013G115200	*SUT*	AT1G22710	CAAACCCTAGCCCACTGAGA	GAATGCCCAAGAGTTGCACA	96
	Actin2/7		CCCATTGAGCACGGTATTGT	TACGACCACTGGCATACAGG	97

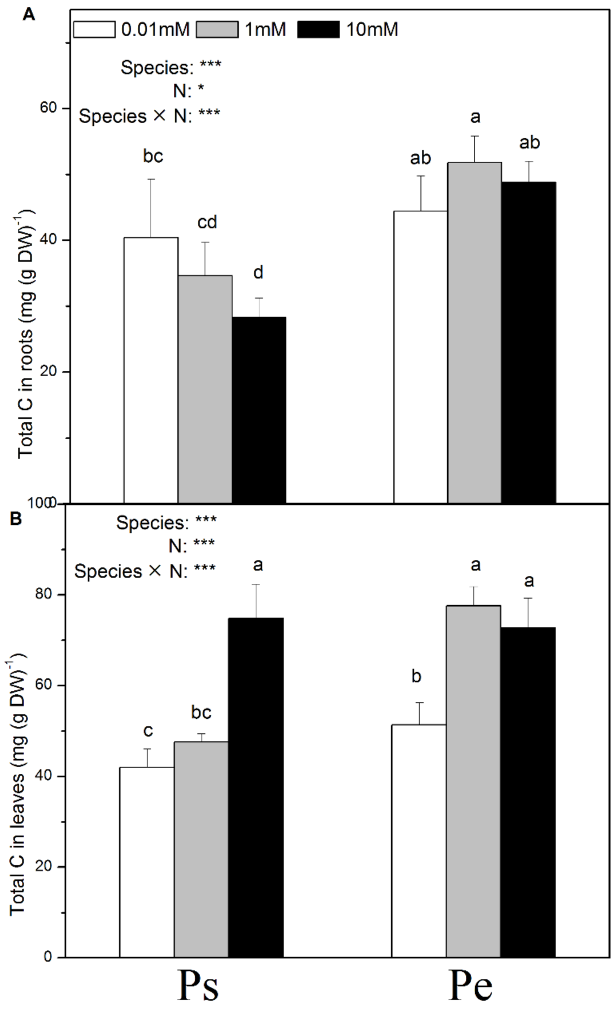

Figure S1. Total C in roots (**A**) and leaves (**B**) of *P. simonii* (Ps) and *Populus euramericana* (Pe) under 0.01, 1 and 10 mM NH_4NO_3. Bars labelled with different letters indicate significant difference between the treatments. *p*-Values of the ANOVAs of species, N treatment, and their interaction are indicated. * $p < 0.05$; *** $p < 0.001$; ns, not significant.

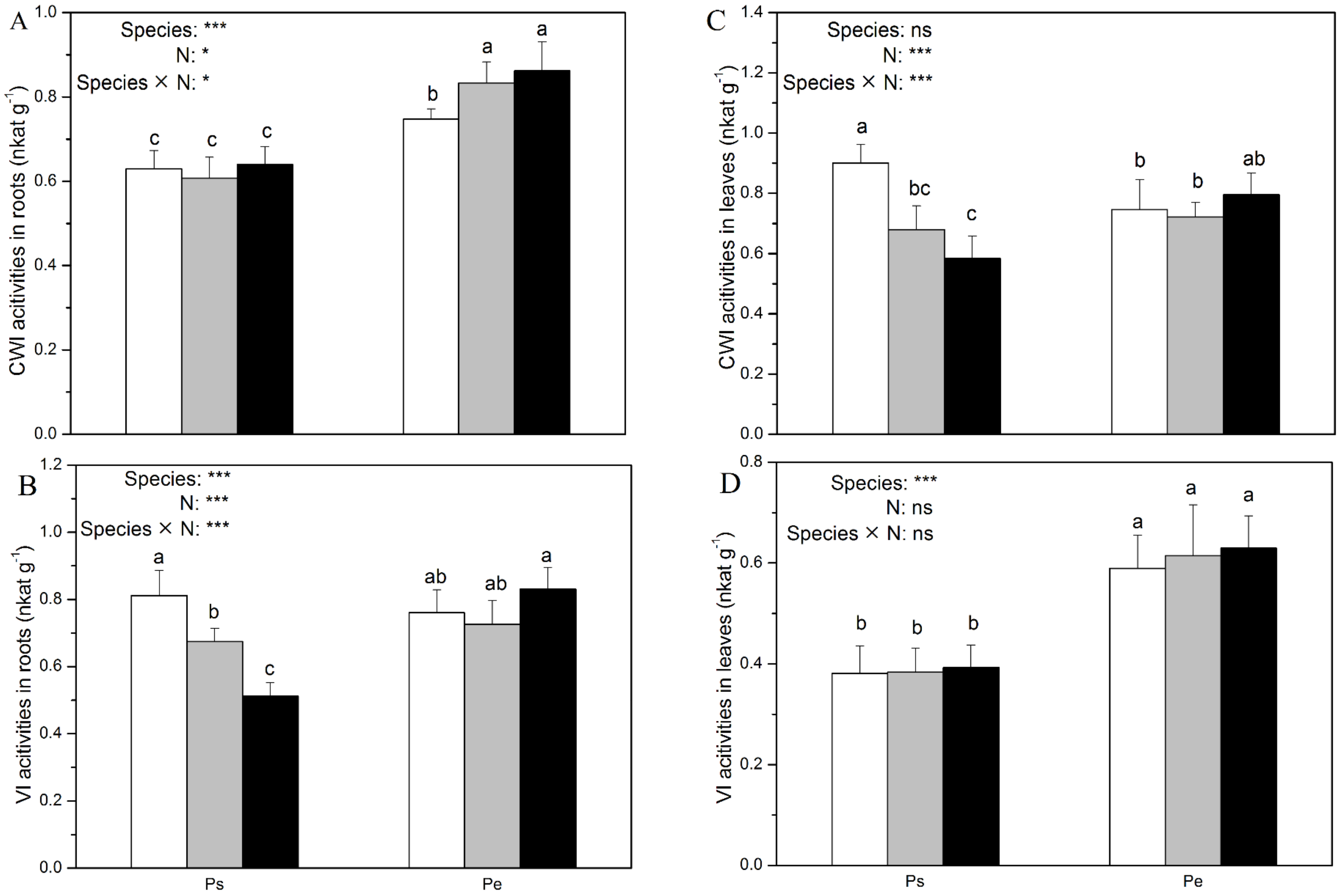

Figure S2. Cell wall invertase (CWI) and vacuolar invertase (VI) in roots (**A**,**C**) and leaves (**B**,**D**) of slow growing P. simonii (Ps) and fast growing P. euramericana (Pe) plants exposed to 0.01, 1 or 10 mM NH4NO3 (N). Bars labelled with different letters indicate significant difference between the treatments. *p*-Values of the ANOVAs of species, N treatment, and their interaction are indicated. * $p < 0.05$; *** $p < 0.001$; ns, not significant.

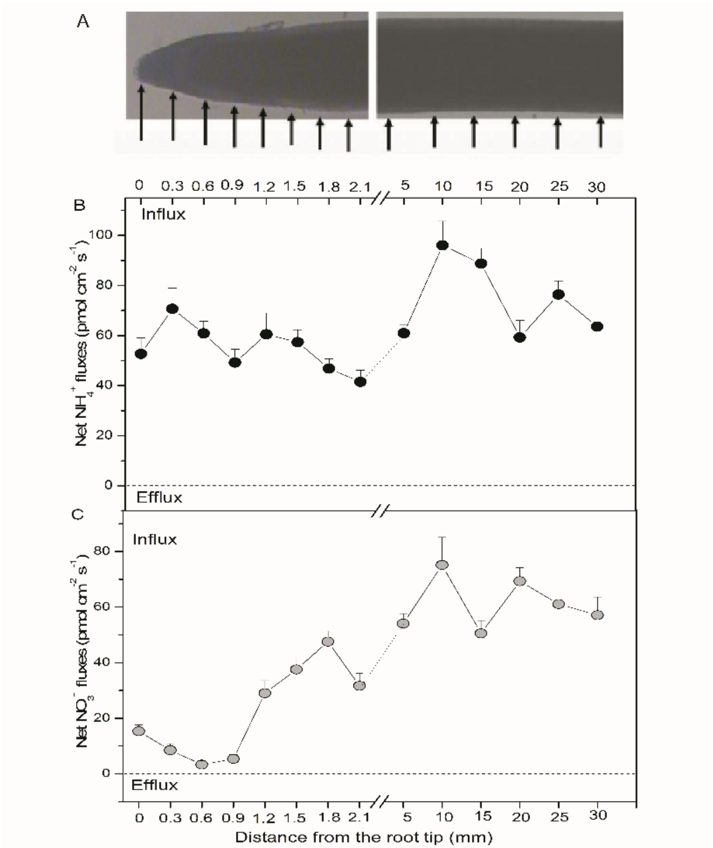

Figure S3. Net NH_4^+ and NO_3^- fluxes along the root of *Populus euramericana* (Pe). For net NH_4^+ and NO_3^- fluxes along the root of *P. simonii* (Ps), see Zhang et al (2014) Net NH_4^+ and NO_3^- fluxes, and expression of NH_4^+ and NO_3^- transporter genes in roots of *Populus simonii* after acclimation to moderate salinity. Trees 28:1813–1821.

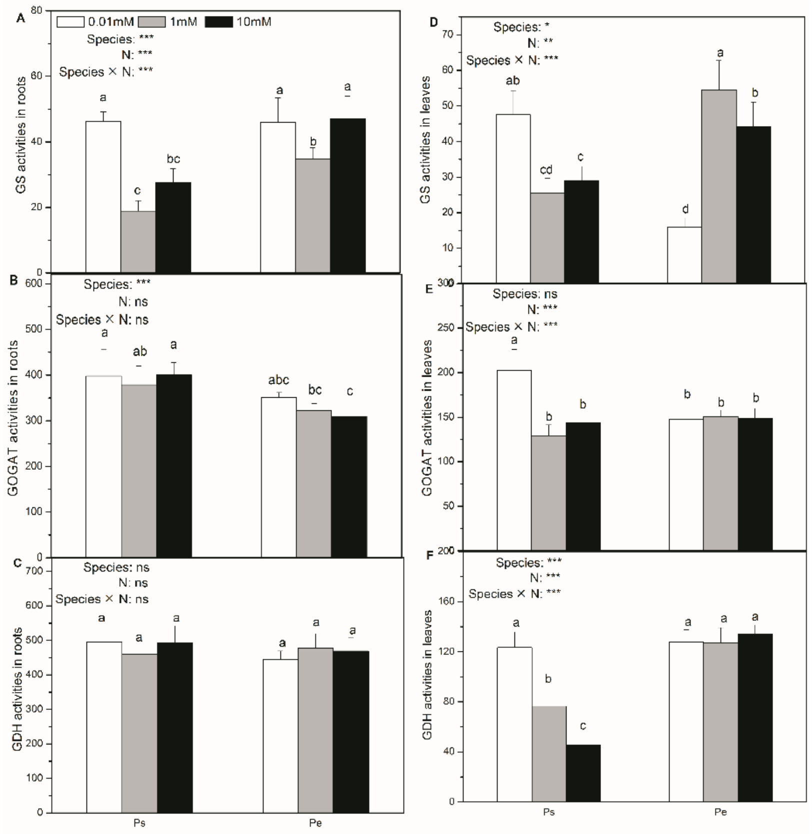

Figure S4. GS, GOGAT, and GDH activities in roots (**A**–**C**) and leaves (**D**–**F**) of slow growing *P. simonii* (Ps) and fast growing *P. euramericana* (Pe) plants exposed to 0.01, 1 or 10 mM NH_4NO_3 (N). Bars labelled with different letters indicate significant difference between the treatments. *p*-Values of the ANOVAs of species, N treatment, and their interaction are indicated. * $p < 0.05$; ** $p < 0.01$; *** $p < 0.001$; ns, not significant.

Table S2. PCA of morphological and physiological responses of *P. simonii* and *Populus euramericana* under 0.01, 1 and 10 mM NH_4NO_3.

Variables	1	2	3	4	5	6	7	8
Root biomass	0.649	0.460	0.330	−0.407	−0.020	−0.018	0.144	0.053
Root length	0.735	0.295	0.087	−0.454	0.158	0.115	0.144	0.047
Root surface area	0.847	0.355	−0.087	−0.239	0.156	−0.038	−0.047	−0.052
Root volume	0.738	0.252	0.192	−0.351	0.006	−0.145	0.050	0.131
Chlorophyll content	0.282	−0.806	0.352	−0.100	0.032	−0.011	−0.084	0.133
Net photosynthetic rate (*A*)	−0.062	−0.341	0.791	−0.054	−0.087	−0.232	0.113	0.058
Stomatal conductance (*gs*)	−0.416	−0.596	0.481	−0.107	−0.301	0.031	0.062	0.067
Transpiration rate (*E*)	−0.624	−0.549	0.457	0.081	−0.060	0.061	0.017	0.150
Root N	0.123	−0.737	−0.270	−0.020	0.227	0.135	−0.018	−0.012
Root ^{15}N	−0.841	0.468	0.125	0.063	0.064	0.006	−0.004	−0.019
Leaf N	0.822	−0.403	0.039	0.148	−0.202	−0.086	0.044	0.019
Leaf ^{15}N	−0.368	0.850	−0.263	0.021	0.053	0.012	0.068	0.006
Root C	0.695	0.448	0.114	−0.151	−0.008	0.002	−0.116	0.183
Leaf C	0.624	−0.494	−0.084	−0.066	0.492	−0.106	−0.015	−0.068
Root nitrate reductase (NR)	0.776	−0.424	0.200	−0.062	0.298	0.010	−0.151	−0.127

Continued:

Leaf nitrate reductase	0.041	−0.309	−0.051	0.280	0.187	0.177	0.696	−0.111
Root nitrite reductase (NiR)	−0.388	0.036	0.399	0.198	0.258	−0.203	−0.178	0.044
Leaf nitrite reductase	0.500	0.145	−0.044	0.431	−0.030	−0.072	−0.096	0.651
Root glutamine synthetase (GS)	0.334	0.671	0.260	0.314	0.130	−0.003	−0.063	−0.324
Leaf glutamine synthetase	0.408	0.000	0.703	−0.318	0.317	−0.073	0.158	0.032
Root glutamate synthase (GOGAT)	−0.743	−0.091	0.105	0.219	0.263	0.082	−0.074	0.165
Leaf glutamate synthase	−0.225	0.509	0.626	0.041	0.285	0.071	−0.067	−0.017
Root glutamate dehydrogenease (GDH)	−0.121	−0.156	0.404	0.057	0.435	0.614	0.054	0.051
Leaf glutamate dehydrogenease	0.561	0.713	0.231	−0.008	−0.093	0.133	0.139	−0.010
Root Ammonioum	0.681	−0.484	0.197	0.126	0.097	0.096	−0.017	−0.210
Leaf Ammonioum	0.624	0.172	0.351	0.475	−0.133	0.147	−0.165	0.089
Root Nitrate	0.620	0.352	−0.219	0.270	0.066	0.214	0.140	−0.252
Leaf Nitrate	−0.096	−0.427	0.013	−0.298	−0.437	0.138	−0.262	−0.393
Root Nitrite	0.769	0.246	0.154	0.105	−0.334	0.029	0.165	0.107
Leaf Nitrite	−0.475	0.759	−0.325	0.085	−0.103	0.039	−0.085	−0.089
NH_4^+ fluxes	0.650	−0.650	0.199	0.237	−0.106	−0.075	−0.033	−0.122
NO_3^- fluxes	0.727	−0.647	0.157	−0.031	−0.093	−0.024	0.000	0.049
H^+ fluxes	−0.566	0.718	0.186	−0.057	0.108	0.003	0.085	0.015
Root Sucrose	−0.527	0.298	0.660	−0.035	0.112	0.009	−0.117	0.046
Leaf Sucrose	−0.182	−0.229	−0.801	0.044	−0.230	−0.114	0.202	−0.008

Continued:

Root Fructose	0.548	0.005	0.510	0.413	−0.247	0.149	0.097	−0.080
Leaf Fructose	0.727	0.218	−0.192	−0.197	−0.038	−0.097	−0.324	0.105
Root Glucose	0.694	0.576	0.117	−0.054	−0.179	0.080	0.162	0.032
Leaf Glucose	0.055	−0.376	−0.307	−0.310	−0.135	0.431	0.335	0.292
Root sucrose phosphate synthase (SPS)	0.822	−0.036	0.032	−0.020	0.262	−0.204	0.074	−0.025
Leaf sucrose phosphate synthase	0.285	−0.564	0.571	0.009	−0.328	−0.081	0.032	−0.063
Root sucrose synthase (SUS)	0.801	0.096	−0.468	0.073	0.122	−0.099	−0.039	−0.066
Leaf sucrose synthase	0.338	0.035	−0.087	−0.278	−0.121	0.605	−0.434	0.020
Root hexokinases (HxK)	−0.446	0.054	0.794	0.035	0.028	−0.058	−0.017	−0.096
Leaf hexokinases	0.765	−0.179	−0.203	0.441	0.106	0.096	−0.114	−0.067
Root cell wall invertase (CWI)	0.900	0.167	−0.042	0.026	0.068	0.018	−0.123	−0.050
Leaf cell wall invertase	0.092	0.581	0.511	−0.145	−0.285	−0.075	0.171	−0.234
Root vacuolar invertase (VI)	0.387	0.631	0.376	0.239	−0.297	0.152	−0.074	0.118
Leaf vacuolar invertase	0.817	0.307	−0.242	0.109	0.055	−0.169	0.089	0.090

Extraction Method: Principal Component Analysis.

a. 8 components extracted.

Table S3. Aerial biomass, intrinsic water use efficiency (WUEi), instantaneous photosynthetic N use efficiency (PNUEi), and nitrogen use efficiency (NUE) of slow growing *P. simonii* (Ps) and fast growing *Populus×euramericana* (Pe) plants exposed to 0.01, 1 or 10 mM NH_4NO_3 (N).

Species	N Supply (mM)	Aerial Biomass (mg DW)	WUEi (mmol CO_2 mol^{-1} H_2O)	PNUEi (mol CO_2 (mg N)$^{-1}$ s^{-1})	NUE (g^{-1} (mg N))
Ps	0.01	7.50 ± 0.39 ^{bc}	24.57 ± 1.13 ^{abc}	4.10 ± 0.11 ^{a}	0.45 ± 0.013 ^{a}
	1	7.24 ± 0.38 ^{c}	19.77 ± 0.88 ^{c}	2.47 ± 0.10 ^{bc}	0.34 ± 0.007 ^{bc}
	10	7.63 ± 0.42 ^{bc}	20.89 ± 1.02 ^{c}	2.79 ± 0.15 ^{b}	0.38 ± 0.016 ^{b}
Pe	0.01	5.25 ± 0.31 ^{d}	23.63 ± 1.94 ^{bc}	1.83 ± 0.13 ^{d}	0.38 ± 0.012 ^{b}
	1	9.09 ± 0.21 ^{b}	29.34 ± 1.92 ^{ab}	2.15 ± 0.08 ^{cd}	0.32 ± 0.004 ^{c}
	10	11.70 ± 0.47 ^{a}	30.10 ± 1.75 ^{a}	2.09 ± 0.15 ^{cd}	0.27 ± 0.011 ^{d}
p-values	Species	***	***	***	***
	N	***	ns	***	***
	Species×N	***	**	***	**

Data indicate mean ± SE (n = 6). Different letters in the same column indicate significant difference ($p < 0.05$). *p*-Values of the ANOVA of drought, nitrogen supply, and their interaction are indicated. ** $p< 0.01$; *** $p< 0.001$; ns, not significant.
